# Perivascular Epithelioid Cell Tumor of the Buttock Region

**DOI:** 10.7759/cureus.15252

**Published:** 2021-05-26

**Authors:** Rafey Rehman, Mariam Aoun, Ronald Levitin, Thomas Quinn, Peyman Kabolizadeh

**Affiliations:** 1 Department of Radiation Oncology, Beaumont Hospital, Royal Oak, USA

**Keywords:** pecoma, perivascular epithelioid cell tumor, soft tissue tumor, mtor, spindle cells

## Abstract

Perivascular epithelioid cell neoplasms, also known as PEComas, are a group of rare mesenchymal tumors that have a perivascular distribution and have no known counterpart to normal cells. The PEComa grouping includes angiomyolipomas, lymphangioleiomyomatoses, clear cell (sugar) tumors at extrapulmonary and intrapulmonary sites, clear cell myomelanocytic tumor of the falciform ligament/ligamentum teres among others. These rare tumors most commonly arise in the uterus. Here, we present an unusual case of malignant PEComa arising in the buttock region.

## Introduction

PEComas are a family of tumors that have a perivascular distribution and are not derived from a counterpart normal cell [1,2]. These rare tumors occur more frequently in women and can appear anywhere from age three to 97 years [3]. PEComas most commonly arise in the uterus, but may also arise in the skin, colon, retroperitoneum, and liver/falciform ligament [3]. PEComas have the capacity to metastasize to adjacent lymph nodes and organs, and the most common organ for metastasis is the lung [3].

Although PEComas are a family of different tumors, they share cellular and structural features. Histologically, the cells noted in PEComa have an epithelioid appearance with acidophilic cytoplasm and are immunoreactive for melanocytic markers (except S100) and sometimes the smooth muscle marker desmin [4,5]. The cells are present around the perimeter of the blood vessel and can infiltrate the smooth muscle layer of the wall [1]. Although these tumors are commonly benign, they may occasionally behave malignantly, depending on the specific location.

## Case presentation

**Figure 1:**
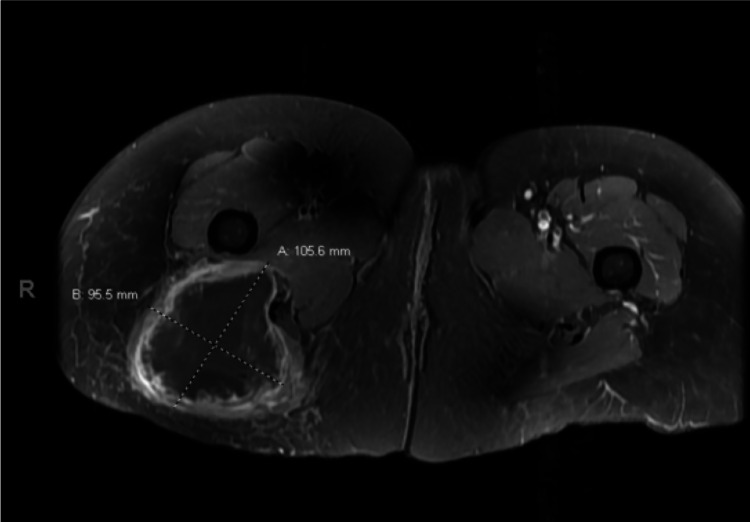
T1-weighted magnetic resonance imaging of the pelvis T1-weighted MRI of the pelvis demonstrates a round, predominantly hypointense lesion measuring 10 × 10 × 11 cm^3^ within the right gluteus maximus muscle. There is predominantly peripheral enhancement and internal T1 hyperintense debris/septation, suggesting central necrosis and internal hemorrhage.

A 49-year-old female with no significant past medical history presented to her primary care physician with a chief complaint of a two-month history of an enlarging mass on her right buttock and discomfort with sitting. The patient denied additional symptoms, including weight loss, fever, and chills. Physical examination revealed a large mass on the right posterior buttock and hip that was mildly tender to palpation. Computed tomography (CT) scan of the right hip was subsequently obtained, which revealed an 8 × 8 × 10 cm^3^ necrotic mass involving the gluteus maximus with preserved fat planes. She was then referred to orthopedic oncology, which recommended magnetic resonance imaging (MRI) and open biopsy. The MRI two weeks after the initial CT scan revealed a large infiltrating soft tissue mass with central necrosis and thick peripheral rim enhancement within the right gluteus maximus, measuring approximately 10 × 10 × 8.5 cm^3^ with minimal preservation of fat planes (Figure 1).

She then underwent open biopsy with pathology revealing high-grade malignancy composed of very atypical epithelioid and spindle cells. The spindle cells were arranged in vague fascicles and showed significant nuclear pleomorphism. Epithelioid cells had clear to eosinophilic cytoplasm and were arranged in nests surrounded by thin capillaries. Neoplastic cells were positive for smooth muscle actin, MART1, EMA, and CD99 immunostains, while negative for WT1, desmin, AE1/3, p63, EMA, CD34, HMB45, GFAP, S100, and SOX10. ERG was patchy positive. Retained nuclear expression was also seen on the INT1 immunostains.

Staging positron emission tomography (PET)/CT scan revealed a small opacity in the left upper lobe of the lung, adjacent to the aortic arch, measuring 8 mm with a max SUV 2.6. There was also a note of the primary tumor in the right gluteus maximus was highly ^18^F-fluorodeoxyglucose (FDG)-avid with a maximum SUV 17.3. The PET interpretation suggested possible inflammatory findings in the lung and treatment proceeded for the primary disease site.

**Figure 2:**
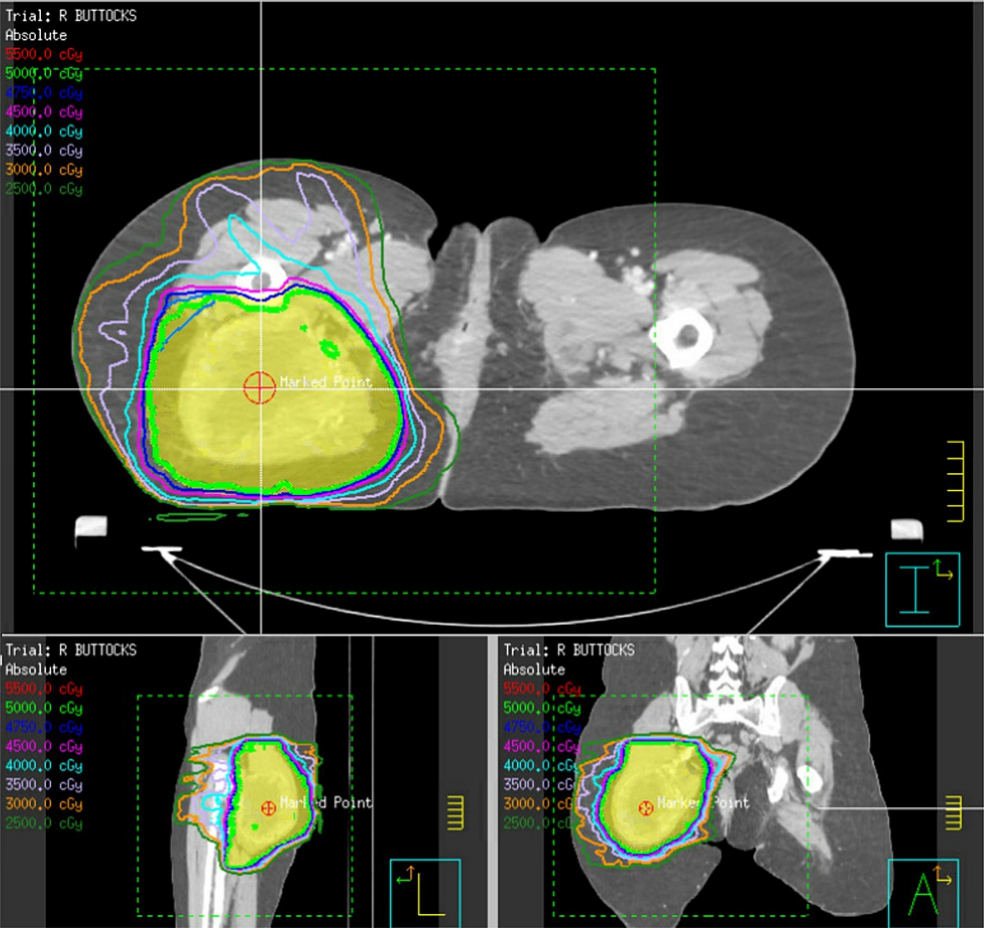
Radiation oncology treatment plan The radiation treatment plan entailed neoadjuvant external beam radiation therapy with 50 Gy that was delivered in 25 fractions to the right buttock region.

Due to concern for margin status and proximity of the tumor to the sacral nerve, the patient was treated with neoadjuvant external beam radiation therapy of the primary tumor with 50 Gy delivered in 25 fractions (Figure 2).

Three weeks after completion of pre-operative radiation therapy, MRI demonstrated T1 hyperintensity with internal debris/septations likely representing internal hemorrhage. The lesion mildly protruded through the sciatic foramen with mass effect, without invasion of the sciatic nerve, adductors, and proximal hamstring musculature without evidence of invasion or nerve enhancement. No adjacent osseous erosion or enhancement was identified. Overall, the mass was noted to have slightly increased in size, measuring 10.6 × 9.6 × 11.1 cm^3^.

She underwent uncomplicated surgical resection with radical resection of the soft tissue mass four weeks following the completion of radiation. The final pathology was consistent with the pre-operative pathology from the open biopsy and confirmed malignant perivascular epithelioid cell tumor (PEComa), measuring 11.7 cm. Histologically, the tumor was comprised of areas with nested cells with pleomorphic round nuclei without prominent nucleoli and clear to finely granular pink cytoplasm as well as larger areas showing markedly pleomorphic nuclei with abundant mitoses. Perivascular growth is present in the original biopsy. Necrosis was present over approximately 50% of the biopsy specimen. Immunohistochemical stains showed the tumor to be positive for the melanocytic marker MART-1, HMB45, and a smooth muscle marker (smooth muscle actin), while negative for other melanocytic markers, including SOX-10 and S100, and a muscle marker (desmin). Overall, it was determined to be a poorly differentiated high-grade pleomorphic sarcoma, but the combination of myomelanocytic immunohistochemical markers as well as the morphology in the lower grade areas confirmed a diagnosis of malignant PEComa, and the findings were consistent with Fédération Nationale des Centres de Lutte Contre le Cancer (FNCLCC) grade 3 disease.

The margins were negative, with the lateral margin (at femur) closest at 1 mm and the deep margin (at sciatic nerve) 2 mm. The proximal margin was 4 mm with all remaining margins >1 cm. She recovered well post-operatively with only mild paresthesia in the area of the surgery.

Repeat imaging with CT chest/abdomen/pelvis two weeks post-operatively revealed enlargement of the previously seen left upper lobe nodule with interval development of multiple diffuse bilateral lung nodules. There was further progression on an outside CT angiogram performed a month later when she presented with increasing dyspnea and hemoptysis. She was seen by hematology/oncology, which recommended systemic chemotherapy with the mechanistic target of rapamycin (mTOR) inhibitor temsirolimus due to metastatic disease, which was initiated once she recovered from a prolonged hospitalization.

Interval surveillance imaging revealed mixed response with substantial improvement in the intrathoracic disease burden, but the progression of disease elsewhere with new evidence of hepatic and osseous metastases with disease located in the T10 vertebral body and in the right sacrum. Outpatient MRI was ordered; however, prior to being performed, she was presented to the emergency department with acute onset of pain in the sacrum after bending down. MRI of the spine revealed new compression fractures at T10 and L4. She underwent corpectomy and kyphoplasty at L4 with radiofrequency tumor ablation and T9-T11 laminectomy with tumor resection.

After recovery from her surgery, she was recommended to undergo postoperative radiotherapy to the two sites in her spine and change therapy to Gemcitabine/Docetaxel; however, she sought an opinion at an outside institution, at which it was recommended to continue the mTOR inhibitor without postoperative radiation. Two months later, she developed severe back pain, and an MRI of the thoracic and lumbar spine revealed recurrent disease at T10 and L5 with the overall progression of the disease. The patient decided to continue seeking care at an outside institution and was therefore lost to follow-up.

## Discussion

PEComas are a family of rare tumors that have a perivascular distribution and occur more commonly in women, but can appear at any age [3]. PEComas most commonly arise in the uterus, but may also arise in the skin, colon, retroperitoneum, and liver/falciform ligament, as well as the buttock region, as demonstrated by our patient case [3]. PEComas have the capacity to metastasize to adjacent lymph nodes and organs, and the most common organ for metastasis is the lung [3]. To the best of our knowledge, this is the only reported instance of PEComa in the buttock region to present with symptoms of discomfort.

The tumor may behave in a benign or malignant fashion, depending on location [2]. For example, while most primary cutaneous PEComas are considered benign, there have been a few reported malignant primary cutaneous cases [2]. However, there have also been cases in which the PEComas neither entirely meet the criteria of malignant or benign tumors. Instead, they have only a subset of the features classic of malignant tumors - such as nuclear pleomorphism or multinucleated giant cells -and have thus been classified as having “uncertain malignant potential” [6]. In our case, the tumor specimen contained spindle cells that show significant nuclear pleomorphism arranged in vague fascicles and epithelioid cells containing clear to eosinophilic cytoplasm that were arranged in nests surrounded by thin capillaries.

The best treatment strategy for PEComas is not yet clear due to the limited number of cases in the literature and the type of metastatic potential an individual case holds. For benign PEComas, surgical excision of the diseased area appears to have the greatest efficacy [2]. However, for tumors that have already metastasized or are in a difficult anatomic location to resect, neoadjuvant therapy, chemotherapy, or radiotherapy have also been used with varied results [3]. The current guidelines of the National Comprehensive Cancer Network (NCCN) recommend the use of the mTOR inhibitors such as sirolimus, everolimus, or temsirolimus for systemic therapy [7]. Unfortunately, there are no specific guidelines on the use of surgery and radiation therapy in the treatment of PEComas, only for the management of soft tissue tumors in general. However, physicians often extrapolate from the literature on soft tissue sarcomas in the management of PEComas, which would entail surgery and radiation therapy for local disease and mTOR inhibitors for metastatic or advanced disease [8]. Neoadjuvant therapy has been used in a small number of cases, with only one reported case of robust objective response following treatment; the remaining cases showed minimal evidence of efficacy [3]. Chemotherapy strategies consist of a wide variety of different possible regimens that share an anthracycline backbone [3]. Interestingly, patients who receive chemotherapy experience a higher rate of recurrence and death, but this is more likely attributable to the advanced progression of the disease in the treated patients than to the actual treatment [3]. In a review by Bleeker et al., two of the eight patients who received adjunct radiotherapy experienced a local recurrence of the disease several months later, so the efficacy of radiotherapy needs to be further explored [3]. Interestingly, for a subset of patients that have a history of tuberous sclerosis and PEComa, a targeted therapy involving mTOR inhibitors-such as rapamycin-can be used, with significant responses in the few cases reported [9-11]. This treatment works because patients with tuberous sclerosis frequently have a mutation in TSC2, which leads to mTOR-mediated tumorigenesis [11]. We also used this treatment strategy, as an mTOR inhibitor was administered to the patient in this case.

Finally, early diagnosis and consequent treatment of PEComas is essential to improve the quality of patients’ lives and outcomes. Many patients with soft tissue tumors tend to present once the mass has reached a significant size to cause symptoms. This delay in symptom presentation can result in patients presenting with metastatic disease at diagnosis. Consequently, non-invasive methods to diagnose the disease earlier during its course may facilitate better clinical outcomes by addressing the primary lesion early. Future studies also need to be conducted on the efficacy of combined treatment strategies, and new treatments altogether as a standard of care to guide treatment decisions are still warranted.

## Conclusions

Perivascular epithelioid cell tumors, or PEComas, are a group of mesenchymal neoplasms that have a perivascular appearance on histology. They are often benign tumors, although they can be malignant in some instances and may show evidence of distant metastasis. Although they most commonly arise in the uterus, it is also important to be aware of other potential locations, such as the skin, liver, colon, and retroperitoneum. The case presented here is unique in that it is the only case of a PEComa occurring in the buttock region to our knowledge based on the literature review. The case also highlights the importance of early diagnosis of this tumor, as metastasis to other organs, such as the lungs and spine, may lead to significant morbidity. Due to the rarity of these tumors, the effectiveness of different modes of therapy has not been thoroughly explored, and larger studies comparing treatment options are certainly warranted.
